# Reply to Kielb et al. Untapped Potential for Female Patients? Comment on “Lucà et al. Update on Management of Cardiovascular Diseases in Women. *J. Clin. Med.* 2022, *11*, 1176”

**DOI:** 10.3390/jcm11113086

**Published:** 2022-05-30

**Authors:** Fabiana Lucà, Furio Colivicchi, Roberta Rossini, Carmine Riccio, Sandro Gelsomino, Michele Massimo Gulizia

**Affiliations:** 1 Cardiology Department, Big Metropolitano Hospital, AO Bianchi Melacrino Morelli, 89129 Reggio Calabria, Italy; 2 Clinical and Rehabilitation Cardiology Department, San Filippo Neri Hospital, ASL Roma 1, 00100 Roma, Italy; furio.colivicchi@gmail.com; 3 Cardiology Unit, Santa Croce e Carle Hospital, 12100 Cuneo, Italy; roberta.rossini2@gmail.com; 4 Cardiovascular Department, Sant’Anna e San Sebastiano Hospital, 81100 Caserta, Italy; carmine.riccio8@icloud.com; 5 Department of Cardiothoracic Surgery, Maastricht University Medical Center, 6229 HX Maastricht, The Netherlands; sandro.gelsomino@maastrichtuniversity.nl; 6 Cardiology Department, Garibaldi Nesima Hospital, 95122 Catania, Italy; michele.gulizia60@gmail.com; 7 Fondazione per ilTuocuore-Heart Care Foundation, 50121 Firenze, Italy

We would like to thank the authors of this letter for their comments [1] on our recently published study [2], which reported several issues that are attracting a lot of interest.

The study of knowledge gaps in risk assessment is a continuously evolving field [3].

Endometriosis is a chronic systemic disease, largely underdiagnosed, that occurs in 10% of reproductive-age females [4]. It has been shown that systemic inflammation, oxidative stress, and endothelial dysfunction are involved in its pathogenesis [5]. Moreover, lower serum levels of high-density lipoprotein, contributing to the atheromatous plaque process, have been reported in endometriosis patients [6,7,8]. An atherogenic lipid condition seems to play an essential role in the vascular injury described in endometriosis [9]. Furthermore, endometriosis has been reported to be strongly associated with hypertension [6]. Surgical treatments such as hysterectomy/oophorectomy, and non-steroidal anti-inflammatory therapies reducing pelvic pain are potentially pathogenic causes of hypertension in endometriosis [6].

The influence of endometriosis on cardiovascular disease (CVD) in women has been recently addressed by anESC consensus document [10], in which the role of female-specific risk factors has been highlighted.

An association between endometriosis, coronary artery disease (CAD), and stroke has also been reported [11,12]. Furthermore, a woman’s reproductive history is thought to be connected with CV morbidity [13,14]. A relationshipbetween CVD and bothnulliparity and grand multiparity (≥5–6 births) has been described [15,16,17], while miscarriage, pregnancy loss (PL), and stillbirth have also been associated with an increased risk of stroke and CVD [18,19].

As reported by Kielb et al., cardiovascular risk assessment remains challenging in women. Being female has frequently been assessed as a predictor of both ischemic and bleeding risks [20]. For example, it has recently been included by the European Society of Cardiology in the SCORE2 risk [21] (a composite model to predict 10-year fatal and non-fatal CVD risk in patients aged 40–69 years without previous CVD or diabetes); in addition, the Academic Research Consortium (ARC) criteria for high bleeding risk (HBR) were evaluated in patients undergoingpercutaneous coronary intervention (PCI) according to sex: in terms of both prevalence and predictive value, females who undergo PCI are more likely to be at ARC-HBR status than males [22].

Notably, the following are the most critical issues to be addressed:

(1) Females with coronary artery disease (CAD) are less likely to receive evidence-based medical and interventional strategies [23,24,25];
(2) The female sex is under-represented in most of the clinical trials [24,26] upon which international guidelines have been based. It has been estimated that CV trials between 2010 and 2017 recruited less than 39% female participants [27];
(3) Female patients are more likely to develop hemorrhagic complications than males [28,29];
(4) Outcomes post-ACS are worse in females [30,31].

There is a higher short-term mortality rate in hospitalized younger women with ST-segment elevation myocardial infarction (STEMI), despite adjustment for medical treatment, primary PCI, and coexisting comorbidities. In particular, women under 60 years of age have an 88% higher risk of 30-day mortality, but this difference is less pronounced after age 60, and disappears in the oldest females [32].

It has been shownthat the median survival time after first myocardial infarction (MI) at ≥45 years of age is 8.2 years for males and 5.5 years for females, while mortality rates at one year are 8% in males and 23% in females [33].

Sex-specific management of NSTE-ACS has not been suggested in either the NICE [34] or the ESC [35] guidelines [27]; on the contrary, the AHA/ACC [36] guidelines suggestan initial conservative approach in low-risk patients with ACS who are troponin-negative, especially women.

However, awareness of the significant disparities in the management of women with CAD is dramatically increasing, and the need for clinical trials specifically focused on women has become more and more evident.

We hope more trials including women, and the application of gender-tailored strategies, will provide different results.
